# Dissociable genetic influences on eye movements during abstract versus naturalistic social scene viewing in infancy

**DOI:** 10.1038/s41598-024-83557-3

**Published:** 2025-02-03

**Authors:** Ana Maria Portugal, Mark J. Taylor, Kristiina Tammimies, Angelica Ronald, Terje Falck-Ytter

**Affiliations:** 1 Development and Neurodiversity Lab (DIVE), Department of Psychology, Uppsala University, Uppsala, Sweden; 2 Center of Neurodevelopmental Disorders (KIND), Centre for Psychiatry Research, Department of Women’s and Children’s Health, Karolinska Institutet and Stockholm Health Care Services, Region Stockholm, Stockholm, Sweden; 3 Department of Medical Epidemiology and Biostatistics, Karolinska Institutet, Stockholm, Sweden; 4 Astrid Lindgren Children’s Hospital, Karolinska University Hospital, Region Stockholm, Stockholm, Sweden; 5 School of Psychology, Faculty of Health and Medical Sciences, University of Surrey, Surrey, UK

**Keywords:** Behavioural genetics, Human behaviour, Attention

## Abstract

Eye-movement metrics like fixation location and duration are increasingly being used in infancy research. We tested whether fixation durations during meaningful social stimulus viewing involve common or different familial influences than fixation durations during viewing of abstract stimulus. We analysed the duration of fixations, and the allocation of fixations to face and motion, from 536 dizygotic and monozygotic 5-month-old twins in: naturalistic scenes including low- and high-level social features, and abstract scenes only having low-level features. We observed significant genetic influences in both conditions (*h*^2^_*naturalistic*_ = 0.30, 95% confidence interval (CI) 0.14 to 0.44; *h*^2^_*abstract*_ = 0.25, 95% CI 0.09 to 0.39), while shared environmental influences were negligible. Although some genetic influences were shared between the two conditions, unique genetic factors were linked to naturalistic scene viewing, indicating that fixation durations index different phenomena dependent on the context. Heritability for face looking was moderate (*h*^2^ = 0.19, 95% CI 0.03 to 0.34), and no familial influences were found for motion looking. Exploratory polygenic score analyses revealed no significant associations with fixation measures. This study underscores the dissociable genetic influences on infants’ visual exploration of abstract versus naturalistic stimuli and the importance of considering context when interpreting eye-tracking data.

**Supplementary Information:**

The online version contains supplementary material available at 10.1038/s41598-024-83557-3.

## Introduction

Visual attention is a key factor in infants’ interaction with their surrounding world, representing their first capacity to interact with the environment by selection of inputs for learning^1–3^. For young infants, eye movements are thought to be predominantly triggered in a bottom-up way by the stimulus characteristics in the environment (e.g., low-level physical salience); and top-down control (by high-level endogenous factors, e.g., familiarity of content, motivation) is limited^4–7^. From three months, top-down control of attention is thought to gradually explain more of infants’ viewing behaviour, leading to more flexible gaze behaviours and active individualized exploration of the visual environment^4–6^. As a result, infants start to differentiate between naturalistic, semantic-rich scenes and those matched in low-level perceptual features but without high-level semantic content, as reflected in differences in their spatiotemporal looking patterns, such as fixation duration^8^. Recent twin studies have been used to study individual differences in eye movements and visual attention in infants and young children^9,10^. These studies have generally found that genetic factors play a substantial role in explaining individual differences in such measures. Further, these twin analyses have indicated that different genetic factors are linked to different types of gaze-based measures, illustrating how these designs can inform us about the unity versus separability of different measures and constructs^11^.

**Fig. 1:**
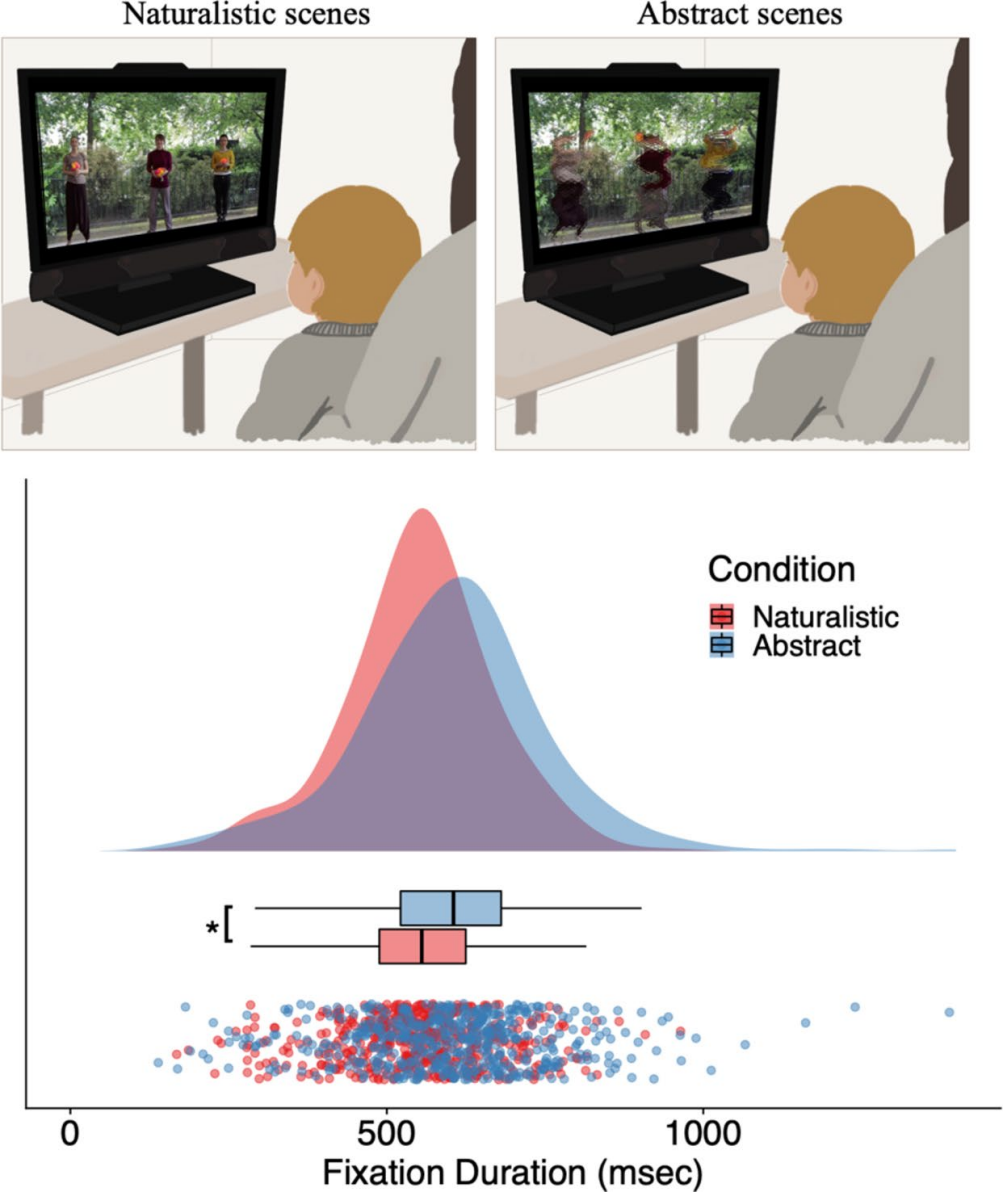
Illustrative set-up and primary fixation duration data plots. Top: Illustration of an infant viewing one video of the naturalistic (top-left illustration) and one video of the abstract (top-right illustration) scenes, illustrations by author A. M. P. . Bottom: Raincloud plots^41^ of the fixation durations derived from each scene, across 536 5-month-old infants (center lines represent the median; box limits represent upper and lower quartile; whiskers represent 1.5× interquartile range; outliers are not presented).

Here, we assessed spontaneous eye movements at 5 months of age in a sample of twins during two experimental conditions (see Fig. 1): naturalistic social scenes, showing dynamic, meaningful activity of human actors; and abstract scenes, showing digitally scrambled versions of the naturalistic stimuli. The abstract scenes were identical to the naturalistic social scenes in terms of low-level features, but they lacked social semantic meaning. We measured the average fixation duration—i.e., the duration of individual looks between fast eye movements (saccades), and the proportion of summed fixation durations on the faces of the actors and on the movements of the actors on the scenes.

A key feature of this experimental design was that it allowed us to assess to what extent fixations were dependent on the stimulus being viewed. Previous work into fixation durations in infancy have typically not investigated to what extent these behaviours are context dependent, but rather treated them as reflecting a unitary underlying process linked to, for example, autism (e.g.^12,13^). Yet, Urabain and colleagues (2017) showed that infants’ mean fixation duration start to be stimulus dependent around 3 to 6 months^8^, which could be explained by attention control mechanisms start shaping viewing behaviour at around this time, e.g. by influences of familiarity of the content. Also, although based on other measures than fixation duration, work on attentional functions in children with autism have found nuanced differences linked to specific types of stimuli^14^.

The infant twin sample consisted of same-sex dizygotic (DZ, fraternal twins) and monozygotic (MZ, identical twins) twins from the BabyTwins Study Sweden (BATSS)^15^, and the twin design allowed us to investigate the etiological factors explaining spontaneous fixation durations to the naturalistic and abstract scenes, and the extent to which these factors were unique or shared between conditions. By comparing the degrees of similarity, within and across phenotypes, in MZ and DZ twins separately, twin studies can be used to quantify the relative contribution of genetic and environmental influences to single phenotypes and the shared and unique influences between phenotypes. Specifically, the variation in a phenotype, or the covariation between phenotypes, is decomposed in a twin model into genetic influences (A, increase twin correlations, more so in MZ), shared environment (C, environmental influences that are shared across twins and increase twin correlations, in the same extent for MZ and DZ), and non-shared environment (E, environmental influences that are not shared between twins and decrease twin correlations).

Based on a preregistered analysis plan (10.17605/OSF.IO/5Q27B), we tested the hypothesis that variability in fixation duration in the naturalistic social scenes condition would be, at least in part, explained by unique genetic factors. This prediction assumed that this condition selectively elicits more top-down processes^8^. Because previous research has indicated genetic influences, and no influence of shared environment, on other looking measures in infants^10,16^, we generally expected genetic influences to be of importance also in the context of fixation durations.

In addition, we tested the genetic and environmental influences to individual variability in fixation allocation to faces and to motion as an additional exploratory analysis of top-down versus bottom-up processing. We also tested the extent to which polygenic risk scores for neurodevelopment (i.e. Autism, ADHD), cognition (i.e. IQ, Educational attainment), and psychopathology (i.e. Schizophrenia, Depression, Bipolar disorder—exploratory) was associated with variation in looking behaviour (duration and spatial allocation) of complex naturalistic scenes; grounded in the hypothesis that genetic predispositions to neurodevelopment conditions and cognitive abilities may influence attentional control and social motivation.

## Methods

### Sample

Participants in this study are part of three hundred and eleven families of same-sex twins recruited to the BabyTwins Study Sweden, BATSS^15^. Families were invited for an initial in-person assessment at 5 months at Karolinska Institutet (data collection from April 2016 to February 2020), and multiple follow-up online questionnaires at 14 months, 24 months, and 36 months. Parents gave informed consent to take part at each time point on behalf of their infants. BATSS was approved by the Regional Ethical Review Board in Stockholm and was conducted in accordance with the Declaration of Helsinki. The main project sample description and inclusion criteria are described elsewhere^15^. Inclusion criteria required same-sex twin pairs, no vision and hearing impairments, no diagnosis of epilepsy or report of seizures, absence of known genetic syndromes or medical conditions likely to impact brain development, and birth at or after 34 weeks of gestation. Zygosity of each twin pair was estimated based on DNA sampled from all infants in the 5-months-visit.

For the current study, a total of 28 twins were excluded due to: parent-reported twin-to-twin transfusion syndrome, report of seizures at the time of birth, very low birth weight (< 1.5 kg), or report of spina bifida. In addition, 23 infants did not complete the eye-tracking assessment due to technical reasons, time constraints, bad calibration, or tiredness. Of the infants that completed this session, 35 infants did not provide good quality data (no or only a few fixations could be estimated), so they were excluded as well. The final sample consisted of 536 twins (285 pairs with at least one twin with data; *n* = 121 DZ Girls, *n* = 134 MZ Girls, *n* = 124 DZ Boys, *n* = 157 MZ Boys), and demographics are summarized in Table 1.

**Table 1:** Descriptive statistics of the main characteristics of the final sample (*n* = 536) split by zygosity. Statistics presented as mean (SD)/min–max. *MZ* monozygotic, *DZ* dizygotic.

	*n*	Overall	Skew	MZ (*n* = 291)	DZ (*n* = 245)
Age (days)^1^	536	167.65 (8.77) 145–203	0.60	167.53 (8.53) 150–194	167.78 (9.07) 145–203
Parent’s age (years)	536	35.21 (4.84) 22.53–49.11	0.29	35.03 (4.76) 22.53–48.86	35.42 (4.94) 23.76–49.11
Parent’s education^2^	536	3.29 (0.74) 1.5–4	− 0.71	3.29 (0.75) 1.5–4	3.3 (0.72) 1.5–4
Term age (days)	536	259.34 (7.37) 238–274	− 0.79	258.31 (7.13) 238–274	260.56 (7.47) 238–274

	N	n MZ	n DZ		
Sex = Female	255 (48%)	134 (46%)	121 (49%)		
Family income^3^ = 1	6	4	2		
2	14	8	6		
3	34	15	19		
4	74	48	26		
5	57	31	26		
6	82	43	39		
7	69	33	36		
8	77	53	24		
9	47	25	22		
10	76	31	45		

^1^5 twin pairs differed in age, in these cases the mean age was used.

^2^Education level was scored on a scale from 1 to 4, where 1 = Primary, 2 = Secondary, 3 = Undergraduate (≤ 3 years) and 4 = Postgraduate level (> 3 years), and averaged across both parents when available.

^3^Family income per month was scored on a scale from 1 to 10, where 1 = < 20 K, 2 = 20–30 K, 3 = 30–40 K, 4 = 40–50 K, 5 = 50–60 K, 6 = 60–70 K, 7 = 70–80 K, 8 = 80–90 K, 9 = 90–100 K and 10 = > 100 K (SEK).

### Eye-tracking

To record infants’ gaze a Tobii TX300 eye-tracker was used (sampling rate of 120 Hz) with Matlab and Psychtoolbox (for stimuli presentation; custom algorithms). An initial 5-point calibration was called before the start of the task battery. This eye-tracking battery involved rotations of free-viewing of the dynamic scenes trials (mixture of social and abstract content, see below), trials of a face pop-out task^10,17^, gaze-contingent gap-overlap trials, pupillary light reflex measurements^18^, and post-calibration sequences; and lasted for about 10 minutes. Infants sat in their caregiver’s lap approximately 60 cm from the presentation screen. Caregivers were instructed before the task to minimize potential influences on the infant’s behavior during the experiment.

The experimental videos used were presented to measure spontaneous looking behaviour to naturalistic and abstract (digitally scrambled) scenes^8^. There were 3 customized naturalistic videos in which three actors alternated between performing baby-friendly actions and 3 abstract videos created from the first set of naturalistic videos (using distortion filters) presented in a fixed order (one group naturalistic-abstract-abstract-naturalistic-naturalistic-abstract, another group abstract-naturalistic-naturalistic-abstract-abstract-naturalistic)—see example frames of the videos in Supplementary Fig. 1. The videos were accompanied by instrumental music. The naturalistic videos have the same dynamics and equal low-level visual features, such as colour or luminance, as the abstract videos, but an added semantic content and familiarity. Videos were presented on a 1310 by 737 pixels rectangle within a screen of 1920 by 1080 pixels (58 cm diagonal). Each video was presented for 21 s. The videos were designed so that three actors occupied distinct regions on the video and alternated between being inactive or active (i.e., moving objects and torso but not moving sides or distance to the camera).

#### Fixation filter

We used the Gazepath R-package^19^ to classify fixations for each infant. This package allows estimation of fixations in infant data based on individual thresholds in a way that noisier data results in more conservative velocity thresholds. Fixations shorter than 100 ms, longer than 2358 ms (95th percentile of the entire sample of fixations in the dataset), and with within-fixation root mean square (RMS) above 1.77 pixels (95th percentile of the entire sample of fixations in the dataset), were excluded. Based on the fixations estimated, the mean fixation duration for naturalistic videos and the mean fixation duration for abstract videos were computed. In addition, measures of data quality for each condition were computed to assess effects of gaze quality in fixation-based measures: number of fixations included when computing the mean duration, mean within-fixation RMS (precision), and proportion of missing data (robustness). See distribution of mean X and Y gaze coordinates across fixations and the mean X and Y gaze coordinates density plots in Supplementary Fig. 2.

#### Spatial fixation allocation analyses

For each frame of the videos (see Supplementary Fig. 3 for an example), each actor Area of Interest (AOI) was coded according to whether the actor was inactive or active, to optimize the dynamic nature of the stimuli and the experimental design. There were frames where all actors were inactive (passive frames), where only one or two were active, and where all were active. An AOI around the face of each woman was drawn with size 350 (center at the face) by 262 pixels (center slightly above face, 1/3 above face center). The % of summed fixation durations in the face AOI when the fixation was on an AOI of an inactive actor, relative to the sum of fixations on AOIs of an inactive actor, in the naturalistic videos was computed to derive the proportion on Face AOI. This deviated from our pre-registration (an ellipse with center on the center of the face) because it ensured we captured face looking even amid noisy infant data while restricting measurements to moments when the actors were inactive to avoid capturing fixations on moving objects in the periphery of the face. The % of summed fixation durations in an active actor AOI, relative to the total sum of fixations that happened in frames where at least one active and one passive actor were present was computed to derive the proportion on Active AOI.

Measures for a condition were excluded if missing data was higher than 95th percentile of the entire sample (45.6% for Naturalistic condition and 47.6% for Abstract) and number of fixations was less than 5th percentile (5 fixations for Naturalistic, and 3 for Abstract). For the observations relative to the AOI measures, measures were additionally excluded when the proportions were exactly 0 or 1 (*n* = 24 in the case of the proportion on Face AOI, *n* = 18 in the case of the proportion on Active AOI).

Gaze quality (number of fixations, RMS, Proportion missing data) was regressed out from measures if significant, before twin and association analyses. Number of fixations, RMS, and Proportion missing data was significantly associated with Fixation Duration in the Naturalistic condition and in the Abstract condition; Number of fixations was significantly associated with proportion on Active AOI in the Naturalistic condition—see all association results in Supplementary Table 1.

### Polygenic scores

DNA samples were genotyped using Infinium Global Screening Array (Illumina, San Diego, CA, USA). Genotype quality control and processing were done using standard procedures and are described elsewhere^15^. Polygenic scores were calculated based on the most recent and largest genome-wide association studies for IQ^20^, Educational attainment^21^, ADHD^22^, autism^23^, bipolar disorder^24^, major depressive disorder^25^, and schizophrenia^26^. The polygenic scores were calculated using the PRS-CS (polygenic prediction via Bayesian regression and continuous shrinkage priors) method^27^. For the polygenic score analysis, the first 10 principal components of ancestry were included as covariates.

### Analytical approach

An analysis plan for this study was registered in OSF (10.17605/OSF.IO/5Q27B) prior to data cleaning and analysis. R software (version 4.0.0) was used for all data analyses. Age (in days) and sex were always included as covariates in statistical models. All statistical testing were two-sided.

Association analyses were performed using the entire twin sample using Generalized Estimating Equations (GEEs, drgee package^28^) to account for the dependency between twin pairs (i.e., one cluster per twin pair id) in our analysis.

#### Condition effects

Condition effects (within-subject Naturalistic vs. Abstract) were tested with a GEE with individual id (instead of twin pair id, to control for the dependence of the two within-subject measures of the same individual) as a cluster variable. While GEEs do not accommodate two levels of clustering (i.e., individual and twin pair clusters simultaneously), we prioritized this method due to its robustness and its use in previous research with this twin sample^10,16,18^. Because two cluster variables cannot be included in the GEE model, this was done separately for Twin 1 and Twin 2 samples and Beta and p-values were reported for both. Gaze quality covariates were included in these models. In addition, the age effect and the interaction between age and condition were tested in a model with condition, age, and the interaction between condition and age included, separately for Twin 1 and Twin 2 samples.

#### Twin modelling

The OpenMx package^29^ (version 2.18.1 with NPSOL optimizer) with full-information maximum likelihood estimation was used for twin analyses, allowing for partially complete twin pairs (one twin data missing) to be included. By comparing the level of within-pair similarity (correlation between twins) separately for monozygotic twins (MZ; who share 100% of their segregating genetic material) and dizygotic twins (DZ; who on average share 50%) twin models can estimate the relative contribution of genetic and environmental factors to the variation in a phenotype. Further, by comparing cross-trait cross-twin (CTCTs) correlations, i.e., the correlation between one phenotype for one twin and another phenotype for their co-twin, these models can estimate the relative contributions of genetic and environmental factors to the covariation between two phenotypes. The variation or covariation can be decomposed into additive genetic influences (A, heritability, which increase twin correlations, more so in the MZ pairs), non-shared environment (E, environmental influences that are unique to each twin and decrease twin correlations, which include measurement error), and either shared environment (C, environmental influences that increase twin correlations in the same way for MZ and DZ pairs, e.g., family socioeconomic status) or non-additive genetic (D) effects (D and C variance cannot be estimated simultaneously from twin data alone). When the pattern of correlations suggested non-additive genetic effects (MZ correlation more than twice the DZ correlation) a decision was made to report an ACE model rather than an ADE model to our data due to sample size (but see Supplementary Information for the ADE model results).

A bivariate saturated model (which tested for the assumptions of the equality of phenotypic and CTCTs correlations across twin order and zygosity) and a bivariate twin model were fitted between the two fixation duration measures (for each condition). A Cholesky decomposition was used to examine genetic influences on fixation durations in the naturalistic conditions that were either unique to that condition or shared with the abstract condition. The best fitting Cholesky decomposition was reported based on the AIC fit statistic (Akaike information criterion, lower values indicate better model fit, which incorporates information about both explained variance and parsimoniousness), and on non-significance (meaning that there was no decrement in fit compared to the saturated or the genetic model, indexed by the χ^2^ distribution). Twin, phenotypic, and CTCTs correlations were derived from the constrained saturated models, in which means, variances, phenotypic and CTCTs correlations were constrained to be equal across twin order and zygosity.

A bivariate twin model was applied to fixation durations to investigate the extent to which genetic and environmental factors were unique or shared between the Naturalistic and Abstract scene conditions. In the case of the spatial fixation allocation metrics (Proportion on face AOI and proportion on active AOI) analyses focused solely on the Naturalistic condition, so univariate twin models were applied.

Associations between genome-wide polygenic scores for cognition (i.e., IQ, Educational attainment), neurodevelopmental conditions (i.e., Autism, ADHD), and psychopathology (i.e., Schizophrenia, Depression, Bipolar disorder) and variation in spatiotemporal looking behaviour to dynamic scenes were tested with the whole twin sample (i.e., including both twins in a pair, including pairs with one twin missing) using GEEs (with twin pair id as a cluster variable, to control for the dependence between twins0. All measures were scaled so that Beta estimates are standardized.

## Results

Sample descriptive statistics are presented in Table 2 (statistics split by zygosity, for statistics split by sex see Supplementary Table 2), and distribution of fixation durations in the naturalistic and abstract scenes can be seen in Fig. 1. In line with previous research^8^, mean fixation duration in naturalistic scenes (Mean = 553, SD = 116, *n* = 521) were shorter than in abstract scenes (Mean = 603, SD = 150, *n* = 520); *p* < 0.001 for both Twin 1 and Twin 2). There were no effects of age on fixation durations (*p* > 0.25 for both Twin 1 and Twin 2), neither an interaction with condition (*p* > 0.25 for both Twin 1 and Twin 2), neither an association between age and proportion on Face AOI (*p* > 0.25, Beta = − 0.02), contrary to previous reports^8^; but note these studies used a longitudinal design with a wider age range.

**Table 2:** Descriptive statistics of the fixation-based measures for each scene condition split by zygosity. Statistics presented as Mean (SD)/min-max.* MZ* monozygotic,* DZ* dizygotic.

	*n*	Overall	Skew	MZ (*n* = 291)	DZ (*n* = 245)
Naturalistic condition					
# Fixations	521	43.64 (27.65) 5–125	0.52	45.82 (27.78) 5–115	41.07 (27.32) 5–125
Proportion missing gaze	521	0.10 (0.11) 0–0.46	1.25	0.10 (0.11) 0–0.46	0.10 (0.12) 0–0.45
Mean RMS	521	0.66 (0.25) 0.17–1.33	0.11	0.63 (0.26) 0.17–1.33	0.69 (0.24) 0.18–1.28
Mean fixation duration	521	552.53 (115.77) 168.06–964.12	− 0.16	553.40 (112.35) 186.67–964.12	551.50 (119.90) 168.06–908.33
Proportion on face	497	0.60 (0.21) 0–0.99	− 0.52	0.59 (0.22) 0–0.99	0.62 (0.20) 0.05–0.99
Proportion on active	503	0.71 (0.16) 0.08–0.99	− 1.46	0.70 (0.16) 0.08–0.95	0.72 (0.16) 0.14–0.99
Abstract condition					
# Fixations	520	30.58 (20.62) 3–100	0.8	31.98 (20.39) 3–94	28.91 (20.81) 3–100
Proportion missing gaze	520	0.11 (0.12) 0–0.47	1.13	0.11 (0.12) 0–0.47	0.12 (0.12) 0–0.47
Mean RMS	520	0.70 (0.26) 0.17–1.5	− 0.03	0.67 (0.26) 0.17–1.5	0.72 (0.25) 0.19–1.39
Mean fixation duration	520	602.84 (149.87) 138.89–1388.89	0.3	605.84 (146.23) 138.89–1388.89	599.22 (154.37) 209.72–1161.67

### Twin analyses

A bivariate Cholesky decomposition with fixation duration in both conditions was fitted (see model results in Supplementary Table 3). Univariate MZ correlations were higher than DZ correlations for fixation duration in both conditions (fixation duration in naturalistic: *r*_*MZ*_ = 0.31, 95% CI 0.14 to 0.46; and *r*_*DZ*_ = 0.12, 95% CI − 0.06 to 0.28; fixation duration in abstract: *r*_*MZ*_ = 0.28, 95% CI 0.11 to 0.43; and *r*_*DZ*_ = 0.03, 95% CI − 0.16 to 0.22). Results showed genetic influences on both fixation duration in naturalistic (A = 0.30, 95% CI 0.14 to 0.44) and in abstract (A = 0.25, 95% CI 0.09 to 0.39) conditions, with the rest of the variance in fixation durations being explained by non-shared environment.

**Fig. 2:**
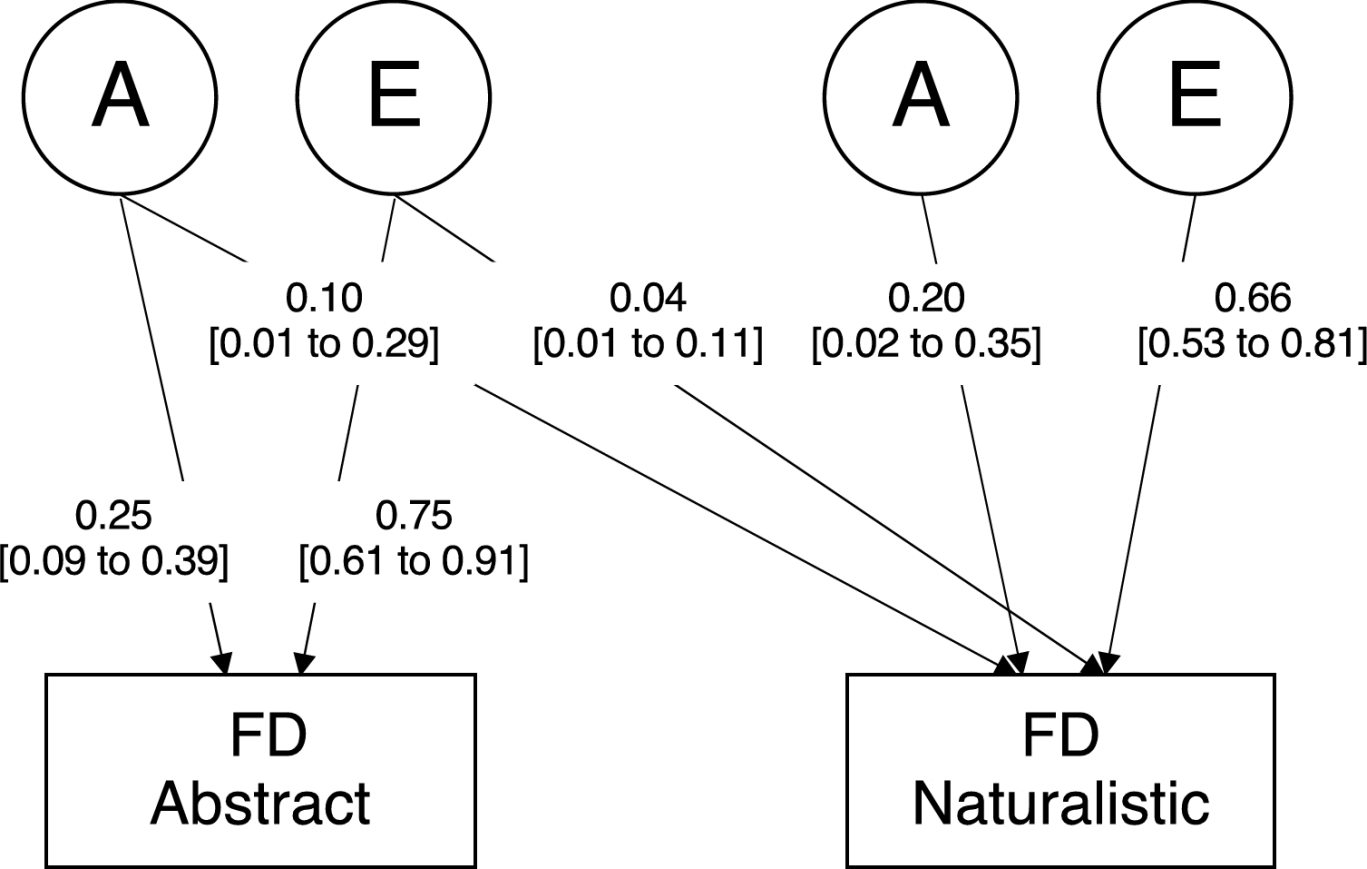
Schematic AE bivariate Cholesky decomposition twin model for fixation durations in the Naturalistic and in the Abstract scenes. Twin structural equation model-fitting was used to decompose the variance in face orienting and face preference into genetic (A) and unique environment (E) influences. Point estimates are shown with 95% confidence intervals in brackets.

The phenotypic correlation between fixation durations in the two conditions was positive and moderate (*r*_*Ph*_ = 0.33, 95% CI 0.24 to 0.41). The cross-twin cross-trait (CTCT) correlations suggested genetic factors could partly explain the association between the two conditions because the MZ CTCT correlation (*r*_*CTCT MZ*_ = 0.17, 95% CI 0.05 to 0.29) was higher than the DZ CTCT correlation (*r*_*CTCT DZ*_ = 0.03, 95% CI − 0.10 to 0.17). Further, The AE Cholesky decomposition (the model with genetic and unique environment influences, and without shared environment influences), reported in Fig. 2, showed significant genetic variation in fixation duration in the naturalistic scenes that were shared with the abstract scenes. Critically, as predicted, it also showed significant unique genetic variation explaining the variance in fixation duration in naturalistic scenes. Most non-shared environment influences (which include measurement error) was linked to each condition separately.

Univariate twin models were fitted for proportion on face AOI and on active AOI (the twin assumptions were met across all measures, see Supplementary Table 4). MZ correlations were descriptively higher than DZ correlations for all measures, suggesting some genetic influence, but for proportion on Active AOI these were very low for both groups (suggesting no familial influences for this measure)—proportion on Face AOI: *r*_*MZ*_ = 0.20, 95% CI 0.04 to 0.35; and *r*_*DZ*_ = 0.04, 95% CI − 0.20 to 0.26; proportion on Active AOI: *r*_*MZ*_ = 0.06, 95% CI − 0.14 to 0.26; and *r*_*DZ*_ = 0.03, 95% CI − 0.18 to 0.23. For proportion on Face AOI, the genetic twin model that best explained the data according to the AIC statistic was an AE model (where A stands for additive genetic effects, and E for non-shared environmental effects); and this showed a heritability of 0.19 (A = 0.19, 95% CI 0.03 to 0.34). While the AE model had the lowest AIC for proportion on face AOI (and was therefore the selected model), other models, such as the CE model, had comparable fit. This suggests some uncertainty in model selection and indicates that the familiarity influences on the proportion on face AOI could also be attributed to shared environmental factors. For proportion on Active AOI, the E model (only non-shared environmental effects) explained the data best. See model fit statistics in Supplementary Table 4.

### Associations with polygenic scores

The associations between the fixation-based measures (FD in naturalistic and in abstract, and proportion on face AOI) and polygenic scores for IQ, educational attainment, autism, ADHD, schizophrenia, depression, and bipolar disorder; were tested and are reported in Supplementary Table 5. There was an uncorrected significant association between the GPS of schizophrenia and FD in abstract scenes (Beta = − 0.13, *p* = 0.031). Applying a Bonferroni multiple comparisons method (alpha threshold/4 = 0.0125), this association was no longer significant. No other significant associations were found.

## Discussion

This study suggests that dissociable genetic factors are involved in eye movement control during infants’ observation of naturalistic meaningful social interaction versus abstract non-social stimuli (with matching low-level properties). Specifically, results showed that individual differences in fixation durations, observed in two distinct, but related, video conditions, were partly genetically dissociated, and only moderately correlated. This suggests that fixation durations measured in different contexts are probably indexing different phenomena and may not generalise well to another one.

Five months is an age where top-down control is rapidly taking over bottom-up-driven gaze behaviour during visual exploration of the environment^4–8^. During observation of meaningful human interaction (naturalistic scenes), observers’ own motivations, preferences, and expectations (endogenous factors) are important determinants of gaze allocation. In contrast, during the abstract scene viewing, gaze is thought to be under influence predominantly of low-level salient physical properties of the stimuli (exogenous factors). Our study indicates that visual exploration across these two viewing conditions were partly genetically differentiated. Although speculative, this may indicate that genetic factors linked to top-down processing are partly independent from genetic factors underlying bottom-up processes. Given that top-down and bottom-up processes are thought to differ in terms of function, development, and brain basis^30^, it is plausible that these processes are supported by partly separable genetic influences early in life in human infants.

The naturalistic scenes included social stimuli (actors performing actions looking directly at the viewer), which means that the endogenous influences at play in this study could be social-specific. Previous adult twin studies have indicated that some components of social cognition (e.g., face recognition) have distinct genetic etiologies, separate from general cognitive abilities^31,32^. The unique genetic influences in the naturalistic scenes condition may be linked to differences in social motivations, preferences, or expectations, which may affect looking tendencies during observation of other people.

While our results could reflect differences in top-down versus bottom-up processing^8^, also other interpretations are possible at this point. First, the observed difference between the two conditions could be driven by exogenously driven face capture present in the naturalistic condition but not the abstract condition. Face capture is fast and automatic, present in newborns, and thought to depend on subcortical processing^33^—thus, the included faces could be seen as salient object influencing attention in a bottom-up manner. However, because we studied five-month-old infants rather than newborns, and measured their gaze during several seconds while they were actively exploring a scene involving complex social interaction (which the face was only a small part of it), a low-level explanation relating solely to social versus physical bottom-up saliency is unlikely in the current case. Second, we cannot assume that performance in the abstract condition was free of any systematic top-down influence. Indeed, the proportion of looking time to the scrambled face area was above chance levels in the abstract condition (but significantly shorter than in the naturalistic condition, see sensitivity analysis I in Supplementary Methods), indicating potential learning effects (i.e., all infants saw all stimuli, it is possible that some understanding of the scenes structure might have come into play during the experimental session). However, while the Abstract condition may have elicited top-down processing, it seems reasonable to assume that it did so to a lesser extent than the Naturalistic scene condition, considering the nature of the stimuli and the young age of the participants. Third, it is possible that the observed genetic differentiation is linked to differential involvement of arousal in the two contexts^34^. Irrespective of the specific nature of the processing behind the observed differentiation, our study shows that fixation duration measures in different contexts cannot be assumed to reflect the same underlying process.

In terms of the spatial attention allocation in the social naturalistic scenes, we found that face looking proportion showed a heritability of 0.19. We have previously reported the heritability of face orienting and preference, studied in static visual pop-out arrays, to be of 19% and 46% respectively^10^. There was no evidence for familial effects (genetic or shared environment) on active/motion looking proportion. Although eye-tracking technology can achieve high spatial precision, infant data are inherently noisy due to compliance variability and motion artefacts. Therefore, we opted for using relatively large AOIs when studying spatial attention allocation to ensure reliable and maximal data capture. We excluded participants with 0 or 1 in the proportion of face looking and active looking to address potential artefacts and outliers; but acknowledge the possibility that this may have removed meaningful extremes in the data distribution. Additionally, restricting face-looking measurements to inactive actors was intended to minimize the misclassification of gaze directed at moving objects (e.g., toys, arms) as face looking. However, this approach limited our ability to examine attention to dynamic faces, which may have provided richer insights into real-world social attention. Future work could benefit from advanced eye-tracking analysis methods and refined naturalistic controlled scenarios to better address these limitations.

While we show that part of the variance in eye movement control is linked to genotypic differences in the infant population, it is important to note that most of the variation was not explained by familial effects in this study. Non-shared environment (E) explained 0.70 (95% CI 0.56 to 0.86) of the variance in the naturalistic scenes, 0.75 (95% CI 0.61 to 0.91) in the abstract scenes, 0.81 (95% CI 0.66 to 0.97) in face looking proportion, and the entire variance in proportion on the Active AOI. Measurement error could be one factor behind this pattern; however, to address this, we implemented several steps to ensure the robustness of our findings, including a thorough data quality check where gaze quality was regressed out from our measures if significant and sensitivity analyses (see sensitivity analysis II in Supplementary Methods) using raw gaze data measurements rather than fixation based ones. E can also reflect real individual differences related to the infant’s mood or behaviour during the session, which can be influenced by situational factors such as illness, feeding, and sleeping arrangements on the day, and the timing of eye-tracking session (which were not conducted simultaneously for both twins). Additionally, it has been shown that infants’ gaze behaviour, both within individuals and stimuli, is generally less predictable and more stochastic than in adults^35–39^, which could contribute to E.

Other limitations of this study must also be considered when interpreting our findings. Specifically, the assumptions inherent in twin models, such as the equal environments assumption (MZ and DZ twins experience similar environments), may not always hold true, which can lead to potential confounding effects on the interpretation of genetic influences and genetic-environment correlations. Additionally, the specific characteristics of our stimuli may limit the generalizability of our results, as the dynamic and experimentally-created nature of the presented scenes and the inherent variability in the visual attention of infants can influence gaze behaviour in ways that might not extend to other contexts.

Regarding the correlations between eye movement control and neurodevelopment/psychopathology related polygenic scores, our results indicated a weak negative association between fixation duration in abstract scenes and genetic susceptibility to schizophrenia, but this result did not survive correction for multiple testing.

Our findings are in line with recent reports indicating that differences in eye movements and gaze behaviour reflect, in part, differences in genetic factors, while shared environment is negligible^10,11,16,18,40^. The current findings extend this emerging field by showing that individual differences in fixation durations are influenced by separable etiological factors dependent on the context. This raises the question of which types of early emerging attentional and perceptual functions can be separated based on their etiologies, how it maps onto findings from brain imaging and behavioural neuroscience, and how it relates to later typical and atypical development.

## Electronic supplementary material

Below is the link to the electronic supplementary material.

Supplementary Material 1

## Data Availability

There was no specification for unrestricted sharing of pseudonymized personal data in the study ethics application, hence data are not available in a public repository. However, data can be requested from Terje Falck-Ytter (terje.falck-ytter@psyk.uu.se). Requests will be responded to within 1 week. Any sharing of pseudonymized (coded) data from the study will require a data sharing agreement according to Swedish and EU law.

## Code availability

The preprocessing and statistical analysis scripts (implemented in R) are publicly available in OSF at https://osf.io/4365h/?view_only=95a2cf363b734acdb1830de0e607afdb. The R code for the raincloud plot visualizations (Fig. 1) has been adapted from Allen et al.^41^.
