# Supplementary material for: Dissociable genetic influences on eye movements during abstract versus naturalistic social scene viewing in infancy
Source: Sci Rep. 2025 Feb 3;15:4100. doi: 10.1038/s41598-024-83557-3 (PMC11791049; doi:10.1038/s41598-024-83557-3)
Supplement: Supplementary file 1 — Supplementary Material 1 [file 41598_2024_83557_MOESM1_ESM.docx]

**Supplementary Information**

**Supplementary Figures**

**
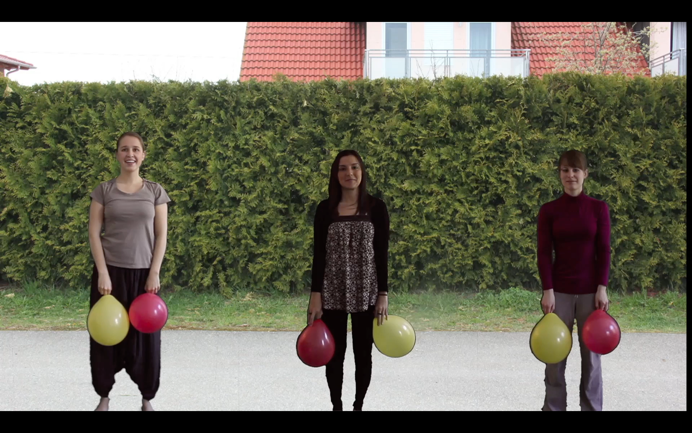

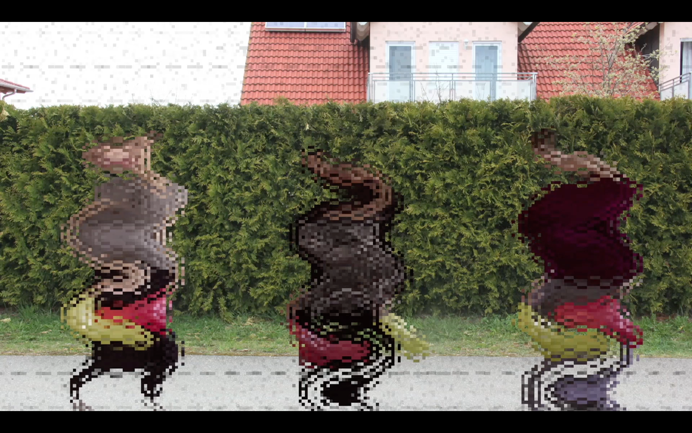
**

**
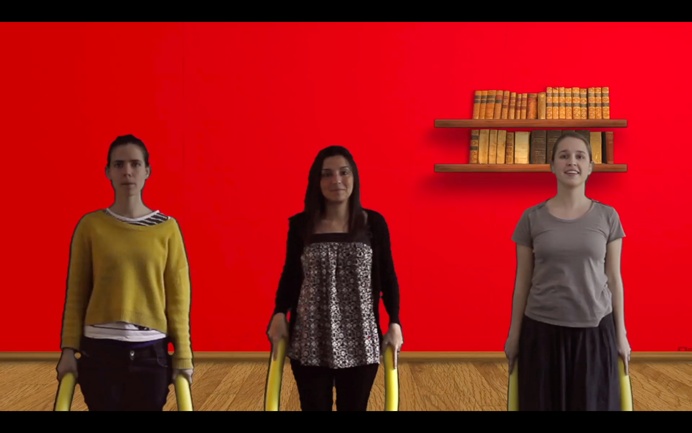

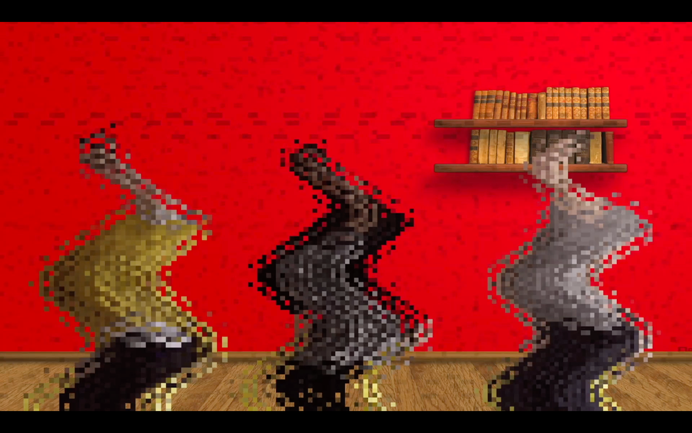
**

**
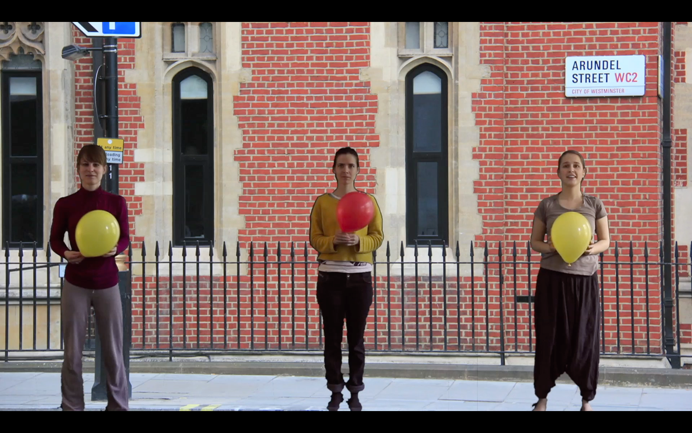

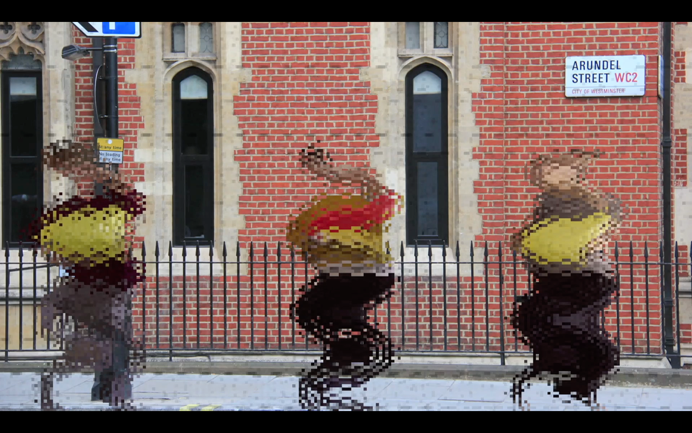
**

**
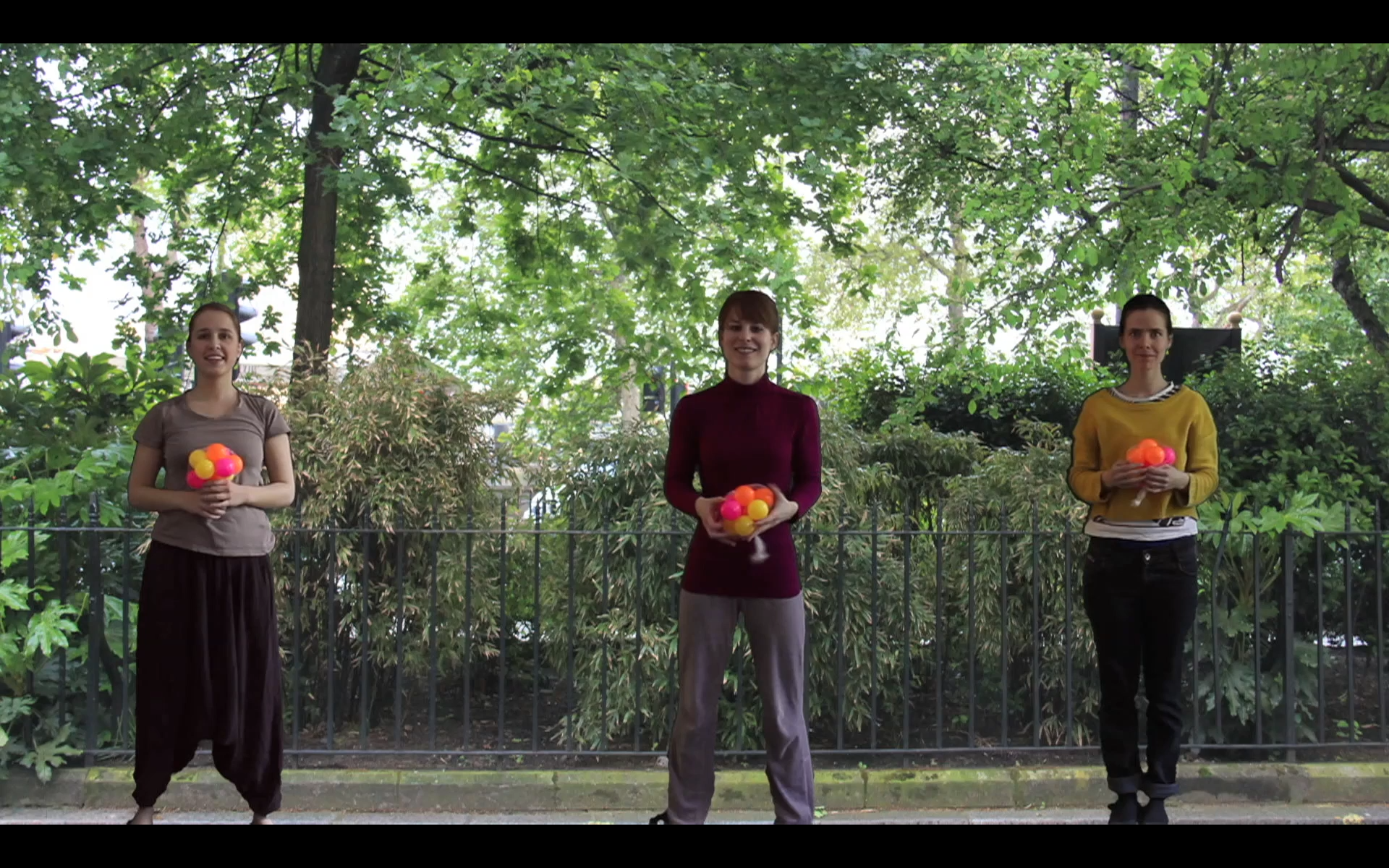

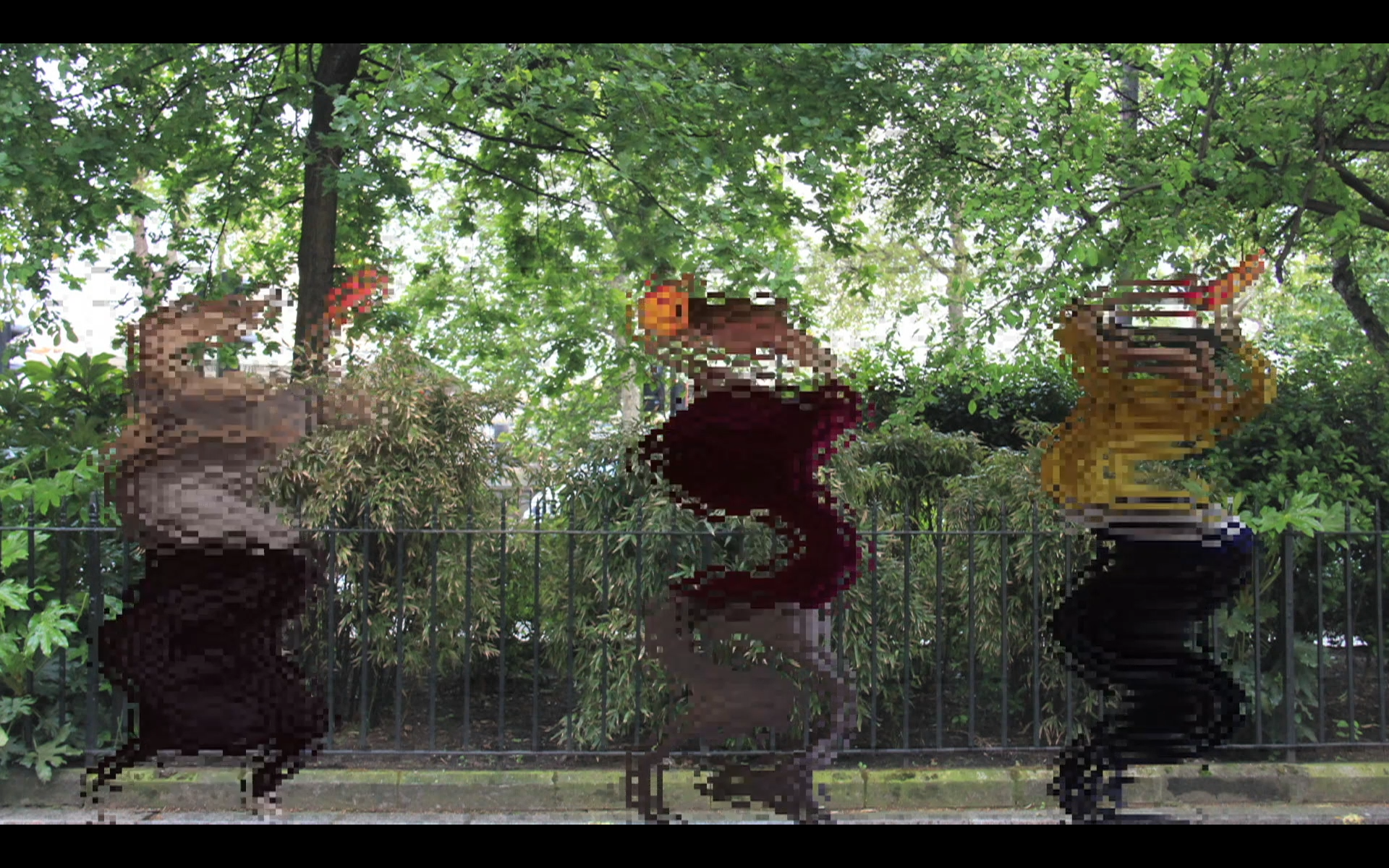
**

Supplementary Figure 1. Example frame per video of the naturalistic condition (on the left) with corresponding video of the abstract condition (on the right). These are stimulus images for which we have obtained the license to use, and no participants or participant data are shown. These experimental videos were created to measure spontaneous looking behaviour to naturalistic and abstract (digitally scrambled) scenes (Urabain et al., 2017)). We thank Tim J. Smith (Birkbeck, University of London) for given permission to use the videos/stimulus set.

**
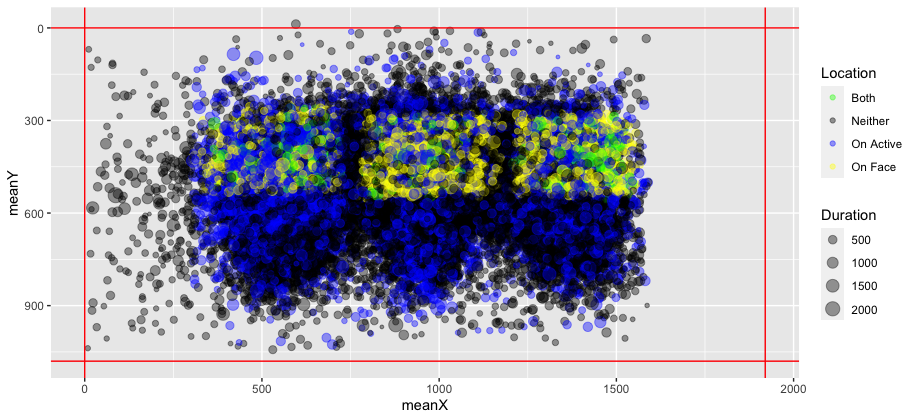

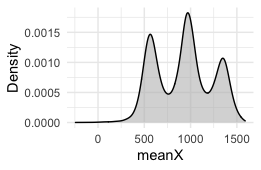

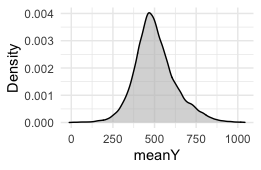
**

*Supplementary Figure 2. Top row: Distribution of mean X and Y gaze coordinates across fixations (n = 40,736). Each point represents a fixation. Vertical and horizontal red lines indicate the screen boundaries. Point size reflects fixation duration, and color represents AOI location. Bottom row: Density plots of the mean X and Y gaze coordinates in arbitrary units.*


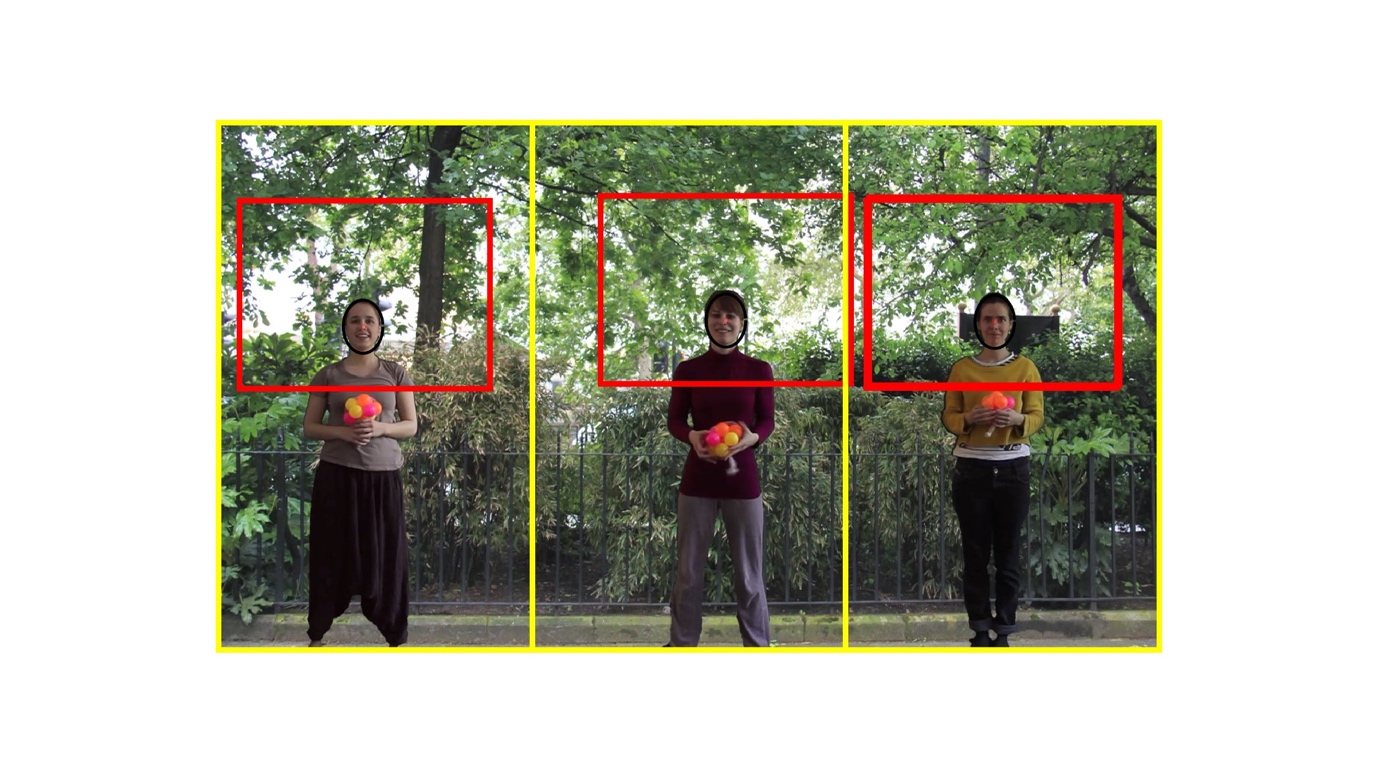


Supplementary Figure 3. One frame of one video of the naturalistic condition) with overlapping AOIs – in yellow women AOI, in red face AOI. This is a stimulus images for which we have obtained the license to use, and no participants or participant data are shown.

**Supplementary Tables**

Supplementary Table 1. Summary of the four GEE models including gaze quality metrics: number of fixations, RMS, Proportion missing data, as predictors of fixation duration in the Naturalistic and in the Abstract scenes, as well as proportion on Face AOI and on Active AOI, with twin pair id as cluster-defining variable. Significant predictors are in bold (alpha threshold was not adjusted for the multiple associations).

|  | Beta Estimate | P-value |
| --- | --- | --- |
| FD Naturalistic |  |  |
| # Fixations in Naturalistic condition | **-1.53** | **< 0.001** |
| Mean RMS in Naturalistic condition | **-85.34** | **0.01** |
| Proportion Missing Gaze in Naturalistic condition | **-574.35** | **< 0.001** |
| FD Abstract |  |  |
| # Fixations in Abstract condition | **-2.00** | **< 0.001** |
| Mean RMS in Abstract condition | **-76.12** | **0.049** |
| Proportion Missing Gaze in Abstract condition | **-601.84** | **< 0.001** |
| Proportion on Face |  |  |
| # Fixations in Naturalistic condition | < 0.001 | 0.853 |
| Mean RMS in Naturalistic condition | 0.11 | 0.053 |
| Proportion Missing Gaze in Naturalistic condition | -0.12 | 0.315 |
| Proportion on Active |  |  |
| # Fixations in Naturalistic condition | **0.002** | **< 0.001** |
| Mean RMS in Naturalistic condition | 0.04 | 0.283 |
| Proportion Missing Gaze in Naturalistic condition | -0.01 | 0.956 |

Supplementary Table 2. Descriptive statistics of the fixation-based measures for each scene condition split by zygosity and sex. Statistics presented as Mean (SD) / min-max. MZ, monozygotic; DZ, dizygotic.

|  | MZ Females  (n = 134) | MZ Males  (n = 157) | DZ Females  (n = 121) | DZ Males  (n = 124) |
| --- | --- | --- | --- | --- |
| Age (days) | 167.67 (9.14)  153-194 | 167.41 (8.01)  150-187 | 167.89 (8.48)  153-189 | 167.67 (9.63)  145-203 |
| **Naturalistic condition** |  |  |  |  |
| # Fixations | 45.18 (26.66)  5-109 | 46.36 (28.78)  5-115 | 41.83 (25.62)  5-114 | 40.32 (28.99)  5-125 |
| Proportion Missing Gaze | 0.09 (0.11)  0-0.43 | 0.1 (0.11)  0-0.46 | 0.09 (0.1)  0-0.36 | 0.11 (0.13)  0-0.45 |
| Mean RMS | 0.66 (0.25)  0.25-1.22 | 0.61 (0.26)  0.17-1.33 | 0.67 (0.25)  0.19-1.28 | 0.70 (0.23)  0.18-1.27 |
| Mean Fixation Duration | 571.42 (102.86)  278.7-832.22 | 537.99 (118.03)  186.67-964.12 | 562.24 (127.02)  168.06-908.33 | 540.83 (111.91)  261.67-814.25 |
| Proportion on Face | 0.59 (0.22)  0-0.99 | 0.59 (0.22)  0.01-0.95 | 0.64 (0.2)  0.12-0.99 | 0.6 (0.2)  0.05-0.97 |
| Proportion on Active | 0.72 (0.14)  0.14-0.95 | 0.68 (0.17)  0.08-0.95 | 0.7 (0.15)  0.14-0. 96 | 0.73 (0.16)  0.16-0.99 |
| **Abstract condition** |  |  |  |  |
| # Fixations | 29.31 (19.33)  3-94 | 34.25 (21.04)  3-92 | 29.75 (20.05)  4-100 | 28.09 (21.58)  3-98 |
| Proportion Missing Gaze | 0.1 (0.12)  0-0.45 | 0.12 (0.13)  0-0.47 | 0.11 (0.11)  0-0.44 | 0.13 (0.13)  0-0.47 |
| Mean RMS | 0.70 (0.25)  0.21-1.5 | 0.65 (0.27)  0.17-1.24 | 0.69 (0.26)  0.19-1.39 | 0.75 (0.25)  0.19-1.28 |
| Mean Fixation Duration | 626.33 (146.17)  138.89-1388.89 | 588.3 (144.46)  169.44-963.54 | 620.84 (152.07)  300-1161.67 | 578.33 (154.32)  209.72-1066.23 |

Supplementary Table 3. Bivariate twin model fit statistics for the fixation durations in the naturalistic condition and in the abstract condition. Best-fitting model in bold.

| Model | # Parameters | -2LL | *df* | AIC | Comparison model | Δ χ2 | Δ *df* | P value | A.1 | A.12 | A.2 | C.1 | C.12 | C.2 | E.1 | E.12 | E.2 |
| --- | --- | --- | --- | --- | --- | --- | --- | --- | --- | --- | --- | --- | --- | --- | --- | --- | --- |
| Fully Sat | 32 | 2856.17 | 1009 | 838.17 | NA | NA | NA | NA | - | - | - | - | - | - | - | - | - |
| 5. | 15 | 2867.38 | 1026 | 815.38 | Fully Sat | 11.21 | 17 | 0.846 | - | - | - | - | - | - | - | - | - |
| ACE model | | | | | | | | | | | | | | | | | |
| ACE | 15 | 2868.59 | 1026 | 816.59 | Fully Sat. | 12.42 | 17 | 0.774 | 0.25 | 0.10 | 0.20 | 0 | 0 | 0 | 0.75 | 0.04 | 0.66 |
| ACE nested models | | | | | | | | | | | | | | | | | |
| AE | **12** | **2868.59** | **1029** | **810.59** | **ACE** | **1.40** | **3** | **1** | **0.25**  **[.09, .39]** | **0.10 [.01, .29]** | **0.20 [.02, .35]** | **-** | **-** | **-** | **0.75 [.61, .91]** | **0.04 [.01, .11]** | **0.66 [.53, .81]** |
| CE | 12 | 2873.46 | 1029 | 815.46 | ACE | 7.37 | 3 | 0.182 | - | - | - | 0.16 | 0.06 | 0.14 | 0.84 | 0.06 | 0.73 |
| E | 9 | 2888.84 | 1032 | 824.84 | ACE | 44.97 | 6 | 0.003 | - | - | - | - | - | - | 1 | .11 | .90 |
| ADE model | | | | | | | | | | | | | | | | | |
| ADE | 15 | 2867.51 | 1026 | 815.51 | Fully Sat. | 11.35 | 17 | 0.838 | 0 | .16 | 0 | 0.27 | 0.10 | 0.05 | 0.73 | 0.03 | 0.65 |

Model definitions. The Fully Sat. model is the fully saturated model of the observed data, which models the means and variances for both variables, and the phenotypic and cross-twin-cross-trait correlations between the two variables, separately for each twin in a pair and across zygosity. **5.** In the Bivariate model fitting, the constrained saturated model equates means, variances, phenotypic and cross-twin-cross-trait correlations across twins within a pair and across zygosity, for both variables of interest.

The best-fitting model (in bold) was the non-significant and most parsimonious model, as well as the one with the lowest AIC.

-2LL = fit statistic, which is minus two times the log-likelihood of the data.

*df* = degrees of freedom

AIC, fit statistic. Lower values denote better model fits.

Δ χ2 = difference in −2LL statistic between two models, distributed χ2.

Δ *df =* difference in degrees of freedom between two models.

A = additive genetic influences

C = shared environment influences

E = non-shared environment influences

A-C/D-E.1 = variance on Phenotype 1 (fixation duration in the abstract condition)

A-C/D-E.12 = variance on Phenotype 2 (fixation duration in the naturalistic condition) that is shared with Phenotype 1 (fixation duration in the abstract condition)

A-C/D-E.2 = unique variance on Phenotype 2 (fixation duration in the naturalistic condition)

Supplementary Table 4. Saturated model and univariate twin model fit statistics and parameter estimates for Proportion on Face AOI and Proportion on Active AOI. Best-fitting model in bold.

| Model | # Parameters | -2LL | *df* | AIC | Δ χ2 | Δ *df* | P value | Comparison model |
| --- | --- | --- | --- | --- | --- | --- | --- | --- |
| Proportion on Face AOI |  |  |  |  |  |  |  |  |
| Saturated Model | 32 | 1151.37 | 1040 | -928.63 | NA | NA | NA | NA |
| Age | 11 | 1396.52 | 486 | 424.52 | 0.43 | 1 | 0.51 | Sat. |
| Sex | 11 | 1397.51 | 486 | 425.51 | 1.42 | 1 | 0.23 | Sat. |
| *Assumptions testing* |  |  |  |  |  |  |  |  |
| 1. | 10 | 1396.63 | 487 | 422.63 | 0.54 | 2 | 0.76 | Sat. |
| 2. | 9 | 1398.47 | 488 | 422.47 | 2.38 | 3 | 0.50 | Sat. |
| 3. | 7 | 1399.30 | 490 | 419.30 | 3.21 | 5 | 0.67 | Sat. |
| 4. | 6 | 1401.93 | 491 | 419.93 | 5.84 | 6 | 0.44 | Sat. |
| *Genetic models* |  |  |  |  |  |  |  |  |
| ACE | 6 | 1402.20 | 491 | 420.20 | 6.11 | 6 | 0.41 | Sat. |
| AE | **5** | **1402.20** | **492** | **418.20** | **0.00** | **1** | **1.00** | **ACE** |
| CE | 5 | 1403.28 | 492 | 419.28 | 1.08 | 1 | 0.30 | ACE |
| E | 4 | 1407.90 | 493 | 421.90 | 5.70 | 2 | 0.06 | ACE |
| ADE | 6 | 1401.94 | 491 | 419.94 | 5.85 | 6 | 0.44 | Sat. |
| Proportion on Active AOI |  |  |  |  |  |  |  |  |
| Saturated Model | 32 | 1151.37 | 1040 | -928.63 | NA | NA | NA | NA |
| Age | 11 | 1413.97 | 492 | 429.97 | 0.16 | 1 | 0.69 | Sat. |
| Sex | 11 | 1414.20 | 492 | 430.20 | 0.39 | 1 | 0.53 | Sat. |
| *Assumptions testing* |  |  |  |  |  |  |  |  |
| 1. | 10 | 1415.20 | 493 | 429.20 | 1.39 | 2 | 0.50 | Sat. |
| 2. | 9 | 1418.82 | 494 | 430.81 | 5.01 | 3 | 0.17 | Sat. |
| 3. | 7 | 1425.03 | 496 | 433.03 | 11.22 | 5 | 0.05 | Sat. |
| 4. | 6 | 1425.03 | 497 | 431.03 | 11.23 | 6 | 0.08 | Sat. |
| *Genetic models* |  |  |  |  |  |  |  |  |
| ACE | 6 | 1425.03 | 497 | 431.03 | 11.23 | 6 | 0.08 | Sat. |
| AE | 5 | 1425.03 | 498 | 429.03 | 0.00 | 1 | 1.00 | ACE |
| CE | 5 | 1425.08 | 498 | 429.08 | 0.05 | 1 | 0.82 | ACE |
| E | **4** | **1425.47** | **499** | **427.47** | **0.44** | **2** | **0.80** | **ACE** |
| ADE | 6 | 1425.03 | 497 | 431.03 | 11.23 | 6 | 0.08 | Sat. |

Model definitions. The baseline model is the fully saturated model of the observed data, which models the means and variances separately for each twin in a pair and across zygosity. **Age.** Testing the significance of age, **Sex.** Testing the significance of sex, **1.** Equating means across twins within a pair, **2.** Equating means across zygosity, **3.** Equating variances across twins within a pair, and **4.** Equating variances across zygosity (i.e., the constrained saturated model).

-2LL = fit statistic, which is minus two times the log-likelihood of the data.

Df = degrees of freedom in degrees of freedom between two models.

AIC, fit statistic. Lower values denote better model fits.

Δ χ2 = difference in −2LL statistic between two models, distributed χ2.

Δ *df =* difference in degrees of freedom between two models.

Supplementary Table 5. Summary of the four GEE models including genome-wide polygenic scores (GPSs) for IQ, educational attainment, autism, ADHD, bipolar disorder, major depressive disorder, and schizophrenia, 10 principal component of ancestry, and age and sex, as predictors of fixation duration in the Naturalistic and in the Abstract scenes, as well as proportion on Face AOI and on Active AOI, with twin pair id as cluster-defining variable. Significant predictors are in bold (alpha threshold was not adjusted for the multiple associations).

|  | Beta Estimate | P-value (uncorrected) |
| --- | --- | --- |
| FD Naturalistic |  |  |
| Age (in days, scaled) | 0.06 | 0.228 |
| Sex (reference level: Female) | **-0.21** | **0.033*** |
| GPS IQ | 0.09 | 0.123 |
| GPS edu. attainment | -0.01 | 0.849 |
| GPS autism | -0.01 | 0.823 |
| GPS ADHD | 0.05 | 0.390 |
| GPS bipolar disorder | -0.01 | 0.947 |
| GPS major depression disorder | -0.03 | 0.614 |
| GPS schizophrenia | 0.01 | 0.882 |
| PC 2 | 4.62 | 0.563 |
| PC 3 | **-11.46** | **0.031*** |
| PC 4 | **26.95** | **0.023*** |
| PC 5 | -3.08 | 0.778 |
| PC 6 | -1.75 | 0.867 |
| PC 7 | 0.04 | 0.997 |
| PC 8 | -8.97 | 0.325 |
| PC 9 | 7.87 | 0.417 |
| PC 10 | -10.86 | 0.257 |
| PC 11 | 5.94 | 0.608 |
| FD Abstract |  |  |
| Age (in days, scaled) | 0.04 | 0.448 |
| Sex (reference level: Female) | **-0.18** | **0.049*** |
| GPS IQ | 0.04 | 0.537 |
| GPS edu. attainment | 0.00 | 0.950 |
| GPS autism | 0.04 | 0.564 |
| GPS ADHD | -0.06 | 0.211 |
| GPS bipolar disorder | 0.04 | 0.594 |
| GPS major depression disorder | -0.01 | 0.831 |
| GPS schizophrenia | **-0.13** | **0.031*** |
| PC 2 | 1.28 | 0.846 |
| PC 3 | -4.82 | 0.288 |
| PC 4 | 8.32 | 0.427 |
| PC 5 | **23.21** | **0.024*** |
| PC 6 | 4.84 | 0.598 |
| PC 7 | -10.85 | 0.319 |
| PC 8 | -2.62 | 0.770 |
| PC 9 | 11.96 | 0.229 |
| PC 10 | -8.40 | 0.390 |
| PC 11 | **19.87** | **0.047*** |
| Proportion on Face |  |  |
| Age (in days, scaled) | -0.05 | 0.365 |
| Sex (reference level: Female) | -0.09 | 0.342 |
| GPS IQ | -0.05 | 0.332 |
| GPS edu. attainment | 0.03 | 0.599 |
| GPS autism | 0.09 | 0.140 |
| GPS ADHD | 0.00 | 0.958 |
| GPS bipolar disorder | 0.06 | 0.453 |
| GPS major depression disorder | 0.01 | 0.883 |
| GPS schizophrenia | -0.07 | 0.291 |
| PC 2 | 1.58 | 0.852 |
| PC 3 | 6.50 | 0.178 |
| PC 4 | 5.68 | 0.575 |
| PC 5 | **-25.97** | **0.016*** |
| PC 6 | -1.54 | 0.886 |
| PC 7 | -2.09 | 0.819 |
| PC 8 | 0.86 | 0.924 |
| PC 9 | 17.84 | 0.108 |
| PC 10 | -0.93 | 0.923 |
| PC 11 | -2.29 | 0.821 |
| Proportion on Active |  |  |
| Age (in days, scaled) | 0.01 | 0.809 |
| Sex (reference level: Female) | -0.10 | 0.275 |
| GPS IQ | -0.04 | 0.473 |
| GPS edu. attainment | 0.00 | 0.945 |
| GPS autism | 0.11 | 0.075 |
| GPS ADHD | -0.03 | 0.595 |
| GPS bipolar disorder | 0.02 | 0.846 |
| GPS major depression disorder | 0.07 | 0.159 |
| GPS schizophrenia | -0.03 | 0.651 |
| PC 2 | 4.09 | 0.536 |
| PC 3 | 4.33 | 0.329 |
| PC 4 | 5.77 | 0.533 |
| PC 5 | -15.65 | 0.124 |
| PC 6 | 14.78 | 0.135 |
| PC 7 | -9.40 | 0.332 |
| PC 8 | 10.57 | 0.227 |
| PC 9 | 11.38 | 0.293 |
| PC 10 | **17.92** | **0.035*** |
| PC 11 | -1.68 | 0.862 |

**Supplementary Methods**

*Proportion on Face and on Active AOI – sensitivity analysis I*

Proportion on AOIs were tested against chance levels, and compared between the two conditions, to make sure the measurements were capturing relevant information.

There was an effect of condition on proportion on Face in a way that proportion was higher on Naturalistic (Mean = .60, SD = .21, n = 497) compared to Abstract (Mean = .47, SD = .23, n = 479), p < .001 for both Twin 1 and Twin 2; suggesting that infants looked more at the face AOI when the face was distinct (i.e., not scrambled). There was no effect of condition on proportion on Active – mean in Naturalistic (n = 503, Mean = .71, SD = .16) and in Abstract (n = 486, Mean = .70, SD = .17) were similar, in Twin 1 p = .09, in Twin 2 p = .99; suggesting infants looked equally to the active AOI (motion) as expected given this was kept the same between conditions.

The proportion of looking on the Face AOI (when in a passive AOI), was significantly above chance level (calculated as .28, face area relative to the stripe area; n = 497, M = .60, SD = .21, one-sample t _Twin 1_(256) = 25.14, p < .001, t _Twin 2_(239) = 22.26, p < .001). Unexpectedly, the proportion on Abstract scenes were also significantly above chance levels, suggesting that infants were looking at the face area even when the face was not distinct.

The proportion on Active AOI was significantly above chance levels. One group of infants in our study (n=368) watched a set of videos (#1, #3, and #4, naturalistic-abstract-abstract-naturalistic-naturalistic-abstract), and another group (n=179) watched another set of videos in which only 2/3 of the videos were the same (#1, #4, and #5, abstract-naturalistic-naturalistic-abstract-abstract-naturalistic). For the first set of videos, the chance value of looking on an Active AOI, calculated by averaging the probability of looking at an active AOI (this probability is ⅓ when only one woman is active and ⅔ when two women are active) for all valid (i.e., at least one active and one passive women) frames, was .38, for the second set the chance value was .41. In both experimental groups, the proportion of looking on an Active AOI was significantly above chance levels (Experimental Group 1, n = 340, M = .70, SD = .16, one-sample *t* _Twin 1_(171) = 24.36, *p* < .001, *t* _Twin 2_(167) = 29.07, *p* < .001; Experimental Group 2, n = 163, M = .71, SD = .16; one-sample *t* _Twin 1_(87) = 16.32, *p* < .001; *t* _Twin 2_(74) = 18.33, *p* < .001). Expectedly, the proportion on Abstract scenes were also significantly above chance levels (*p* < .001 for both Twin groups).

*Proportion on Face and on Active AOI based on raw gaze – sensitivity analysis II*

To guarantee that results were not driven by a poor estimation of fixations in our dataset, we computed proportion on Face and on Active AOIs using sum of raw gaze data in the AOIs instead of fixation data. A measure of data quality – mean looking time in seconds, i.e., the exact opposite of missing data, was also computed. Observations for each condition were excluded if mean looking time was less than 5% percentil (11.3 seconds for Naturalistic, 10.7 for Abstract). Mean looking time was regressed out of from measures, if significant, before main analyses. For proportion on Face in Naturalistic the covariate was not significant; for proportion on Active in naturalistic, the covariate was significant.

The raw-based AOI measures in the Naturalistic scenes were significantly correlated with the fixation-based AOI measures: for proportion on Face (Beta = .84, p <.001) and proportion on Active (Beta = .60, p < .001). The results using raw-based AOI measures were very similar to the ones with the fixation-based measures:

- The proportion on Active AOI in the naturalistic condition, computed with raw data, were significantly above chance levels.
- The proportion on Face AOI in the naturalistic condition, based on raw data, were also significantly above chance levels.
- There was an effect of condition on Face, proportion is higher on Naturalistic (Mean = .59, SD = .18, n = 539) compared to Abstract (Mean = .48, SD = .19, n = 539), p < .001).
- Age was not significant to predict proportion on Face AOI in Naturalistic condition (p > .25).
- The twin models showed generally similar results – the AE model was the best fit for Proportion on Face AOI in Naturalistic, but the CI of the estimate of A included 0 (was .16); the E model was the best fit for proportion on Active AOI.

However, some results differed between methods:

- There was an effect of condition on proportion on Active that there it was not seen in the fixation-based measures: the proportion in Naturalistic (n = 539, Mean = .72, SD = .11) is slightly higher than in Abstract (n = 539, Mean = .67, SD = .12). p < .001 for both Twin 1 and Twin 2. This might be driven by a lower mean in the Abstract condition using the raw-based measures.
